# Pharmacokinetic variability and significance of therapeutic drug monitoring for broad-spectrum antimicrobials in critically ill patients

**DOI:** 10.1186/s40780-025-00425-6

**Published:** 2025-03-17

**Authors:** Ryota Tanaka

**Affiliations:** Department of Clinical Pharmacy, Oita University Hospital, Yufu, Oita Japan

**Keywords:** Critical illness, Pharmacokinetics/pharmacodynamics, Antimicrobials, Therapeutic drug monitoring, Intensive care, Febrile neutropenia

## Abstract

Critically ill patients are susceptible to serious infections due to their compromised conditions and extensive use of medical devices, often requiring empiric broad-spectrum antimicrobial therapy. Failure of antimicrobial therapy in this vulnerable population has a direct impact on the patient’s survival; hence, selecting the optimal dosage is critical. This population, however, exhibits complex and diverse disease-related physiological changes that can markedly alter antimicrobial disposition. Inflammatory cytokines overexpressed in the systemic inflammatory response syndrome increase vascular permeability, leading to higher volume of distribution for hydrophilic antimicrobials. These cytokines also downregulate metabolic enzyme activities, reducing the clearance of their substrates. Hypoalbuminemia can increase the volume of distribution and clearance of highly protein-bound antimicrobials. Acute kidney injury decreases, while augmented renal clearance increases the clearance of antimicrobials primarily excreted by the kidneys. Furthermore, continuous renal replacement therapy and extracorporeal membrane oxygenation used in critical illness substantially affect antimicrobial pharmacokinetics. The complex interplay of multiple factors observed in critically ill patients poses a significant challenge in predicting the pharmacokinetics of antimicrobials. Therapeutic drug monitoring is the most effective tool to address this issue, and is proactively recommended for vancomycin, teicoplanin, aminoglycosides, voriconazole, β-lactams, and linezolid in critically ill patients. To streamline this process, model-informed precision dosing is expected to promote personalized medicine for this population.

## Background

Critically ill patients are at increased risk of developing serious infections due to the extensive use of invasive diagnostic and therapeutic devices including ventilators, intravascular catheters, bladder catheters, extracorporeal membrane oxygenation (ECMO), and continuous renal replacement therapy (CRRT), in conjunction with their compromised physical condition [1]. Severe infections such as bacteremia and febrile neutropenia (FN) potentially lead to systemic inflammatory response syndrome (SIRS), which has implications beyond the local inflammatory response. SIRS is identified based on clinical criteria including fever or hypothermia, tachycardia, tachypnea, and abnormal white blood cell counts indicating either leukocytosis or leukopenia [2]. It serves as an early marker of sepsis, and if not treated promptly, can lead to multiple organ dysfunction syndrome and mortality [3]. Severe sepsis is characterized by organ failure, whereas septic shock is defined by persistent hypotension despite adequate fluid resuscitation, necessitating the administration of vasoconstrictor support. The mortality rate associated with sepsis ranges from 20 to 30%, and the mortality risk is even higher in patients with severe sepsis and septic shock [4]. Several factors contribute to these mortality rates, including demographic variables and underlying diseases [5, 6], the number of failed organs [7], infection site and severity [8], and efficacy of antimicrobial therapy. Given these characteristics, an effective infectious disease management strategy for critically ill patients is pivotal and requires judicious use of antimicrobials at sufficient doses.

Empiric and prompt antimicrobial therapy is an effective strategy for treating critically ill patients [9, 10]. However, outcomes remain unsatisfactory, indicating that dose optimization may be a viable means to improve outcomes in this population [11, 12]. The appropriate dose and administration interval should be determined based on the relationship between the pharmacokinetic (PK) and pharmacodynamic (PD) properties of each antimicrobial agent. The optimal PK/PD parameter depends on the antimicrobial bacterial activity, which is concentration- and/or time-dependent, as follows: (1) peak plasma concentration (Cpeak)/minimum inhibitory concentration (MIC) for antimicrobials with concentration-dependent activity; (2) cumulative percent of time in 24 h that the free drug concentration remains above the MIC (fT > MIC) for antimicrobials with time-dependent activity; (3) area under the concentration-time curve for 24 h (AUC)/MIC for antimicrobials with concentration- and time-dependent activity [13]. However, the disposition of antimicrobial agents in critically ill patients is subject to variability due to multiple interconnected factors. Physiological changes associated with critical illness may affect drug absorption, distribution, metabolism, and excretion. These changes potentially cause significant alterations of volume of distribution and clearance, leading to inadequate antibiotic exposure and increased risk of morbidity and mortality. In addition, extrarenal losses contribute to reduced drug retention in the body, exacerbating the challenge of attaining effective therapeutic levels. A study conducted in intensive care units (ICU) in Sweden reported that 45% of patients did not achieve the PK/PD target of 100% fT > MIC for β-lactam antibiotics during the first 72 h of treatment [14]. This result was comparable to other previous studies reporting 40–45% [15, 16, 17]. Conversely, in critically ill patients, organ damage often occurs as a result of biological factors such as inflammatory cytokines and mediators produced during severe infections and trauma. This, in turn, may lead to antimicrobial overexposure, resulting in concentration-dependent adverse events for certain antimicrobials. Given these complexities, standard dosing regimens may not be sufficient, necessitating dose titration and therapeutic drug monitoring (TDM) to optimize antimicrobial therapy.

This review provides a comprehensive overview of the factors associated with the PK alterations of antimicrobials, particularly those with broad-spectrum activity, in critically ill patients, with discussions on dose optimization according to these alterations and the benefits of TDM.

## Main text

### Factors influencing PK of antimicrobials in critically ill patients

Several pathophysiologic factors change during critical illnesses, which may alter the PK of antimicrobials. Comorbidity of multiple conditions that may affect drug disposition is common and complicates PK prediction. The major critical factors that may influence antimicrobial PK in critically ill patients are: (1) inflammation, (2) augmented renal clearance (ARC), (3) hypoalbuminemia, (4) acute kidney injury (AKI), (5) renal replacement therapy (RRT), and (6) extracorporeal membrane oxygenation (ECMO).

### Inflammation

Inflammation, a body response to harmful stimuli, can lead to SIRS in critically ill patients [18]. This systemic inflammation is prevalent in sepsis, and is characterized by elevated inflammatory markers such as C-reactive protein (CRP), tumor necrosis factor-alpha (TNF-α), and interleukin-6 (IL-6). Severe inflammation significantly impacts drug PK by altering absorption, distribution, metabolism, and excretion [19]. This may result in variability in drug exposure, particularly in critically ill patients, and may require dose adjustment to ensure therapeutic efficacy and avert toxicities. The influence of inflammation on PK is complex and multifaceted, involving various physiological and molecular mechanisms. Inflammation has been reported to alter the volume of distribution of several antimicrobials, and affect penetration into the cerebrospinal fluid (CSF) or peritoneal cavity [20–22]. Inflammatory cytokines can cause damage to the glycocalyx and endothelial cells, thereby promoting extracellular fluid leakage. This may lead to an increase in volume of distribution of hydrophilic antimicrobials such as amikacin [20]. In addition, the CSF concentration of meropenem is increased in patients with elevated IL-6 levels [21], whereas the intraperitoneal concentration of amphotericin B is reduced with increase in CRP level [22]. In addition, acute inflammation inhibits the activity of drug-metabolizing enzymes represented by cytochrome P450 (CYP) enzymes, especially CYP3A and CYP2C19 [23]. This results in decreased drug clearance and increased drug exposure, especially for drugs with low extraction ratios [24]. Inflammation also affects the metabolism of some antimicrobials such as voriconazole, and may lead to overexposure due to altered metabolic activities [25]. Voriconazole exhibits significant inter- and intra-individual PK variability. Factors contributing to this variability include age, liver function, genetic polymorphisms, drug interactions, and, in particular, inflammation [26, 27]. Several studies have demonstrated a positive correlation of CRP level with voriconazole exposure, metabolic ratio, clearance, and dose-normalized trough concentration, both between and within individuals [28–32]. TDM and dose titration considering fluctuation in CRP level may be crucial when using voriconazole.

### Augmented renal clearance

ARC is defined as measured creatinine clearance (Ccr) greater than 130 mL/min/1.73 m^2^, and manifests in 20–65% of critically ill patients [33, 34]. ARC poses significant challenges in maintaining therapeutic drug levels, especially for drugs primarily excreted by the kidneys [33, 35]. Several potential factors may be associated with ARC, such as increased metabolism, alteration in neurohormonal balance, and fluid resuscitation. The risk factors include younger age, male, sepsis, burns, surgery, trauma, subarachnoid hemorrhage, and FN [36–41]. Many antimicrobials such as β-lactams and glycopeptides possess hydrophilic properties and are primarily excreted by the kidneys via glomerular filtration. In patients with ARC, exposure to these antibiotics may be significantly reduced [42, 43]. Wong et al. [44] identified ARC as the strongest predictor of subtherapeutic β-lactam exposure in critically ill patients. A systematic review by Silva et al. [45] identified 47 studies that provided dosing recommendations for 18 antibiotics in critically ill patients. This review demonstrated that most antibiotics required incremental dosing adjustment or infusion modality adjustment to achieve PK/PD targets, underscoring the necessity of dose adjustment of antimicrobials in patients with ARC.

FN, a severe infection, is generally defined by a neutrophil count of 500/µL or less and an axillary temperature of 37.5℃ or higher. This infection can lead to elevated systemic inflammatory cytokine levels and ARC in both adults and children. Indeed, previous studies in pediatric patients with FN reported that more than 50% of the patients met the criteria for pediatric ARC (≥ 160 mL/min/1.73 m^2^) [46–50]. Accordingly, vancomycin clearance was significantly increased in pediatric FN patients compared with non-FN patients [46, 50], and standard intermittent administration of tazobactam/piperacillin resulted in insufficient probability of target attainment in pediatric patients with FN [47]. Moreover, a population PK modeling study demonstrated that the currently approved dosing regimen of tazobactam/piperacillin for pediatric patients with FN (80 mg/kg every 6 h, 30-min infusion) is inadequate to achieve the target PK/PD index [49]. A population PK model of vancomycin in pediatric patients with FN suggests that children aged 6 months to 6 years with ARC require an initial vancomycin dose 35–65% higher than current dosing recommendations [51]. Because clearance of hydrophilic antimicrobials such as vancomycin and β-lactams is enhanced in ARC, resulting in subtherapeutic exposure, dose escalation would be required to ensure therapeutic efficacy.

### Hypoalbuminemia

Protein binding rate is a crucial property of drugs, referring to the extent of their binding to proteins in blood. This parameter is important because only free drugs can penetrate the tissues and organs to exert both efficacy and potential adverse effects. Several antimicrobials used in critically ill patients have high protein binding rates, including ceftriaxone, ertapenem, tigecycline, teicoplanin, daptomycin, tedizolid, micafungin, caspofungin, and posaconazole [52–54]. Hypoalbuminemia is common in critically ill patients, especially those admitted to the ICU, with reported incidence as high as 40–50% [52]. Because of its association with increased morbidity and mortality, hypoalbuminemia is a major concern in the management of critically ill patients. It also has significant impact on the disposition of antimicrobials, particularly those with high protein binding rates. Low plasma albumin levels can alter the binding affinity of highly protein-bound drugs, temporarily elevating free drug concentrations in the circulation. This phenomenon can improve therapeutic efficacy but also increase the risk of toxicity, particularly for drugs with a narrow therapeutic window. However, if the organ function involved in drug elimination is preserved, the free fraction is immediately diluted by increased total body fluids after administration, and then subsequently rapidly eliminated. Consequently, hypoalbuminemia reduces the total concentrations of highly protein-bound drugs while potentially not altering the free concentrations that are associated with efficacy and toxicity. Based on this theory, clinical practice guidelines for teicoplanin TDM issued a weak recommendation to lower target trough concentrations depending on serum albumin level [55]. Moreover, both the volume of distribution and clearance of highly protein-bound antimicrobials such as ceftriaxone, ertapenem, teicoplanin, aztreonam, fusidic acid, and daptomycin are significantly increased in critically ill patients with hypoalbuminemia, potentially leading to underexposure and failure to achieve desired PD indices [52]. Therefore, therapeutic strategies such as higher loading doses or more frequent dosing schedules should be carefully reviewed to maintain adequate therapeutic levels throughout treatment [56]. Future studies specifically focused on tailoring dosing to the unique PK profile seen in individuals with low serum albumin levels are warranted, and could be extremely helpful in optimizing outcomes and minimizing adverse effects in this population.

### Acute kidney injury

AKI is characterized by a sudden loss of kidney function, resulting in accumulation of waste products in the body. It is particularly prevalent among critically ill patients, with incidence of up to 50% in ICU patients [56]. Common AKI triggers include sepsis, major surgeries such as open heart surgery, and acute decompensated heart failure [57]. According to the KDIGO guidelines, the diagnosis and staging of AKI are determined by an increase in serum creatinine from baseline and a decrease in urine output [58]. The impact of AKI on the PK of antimicrobials primarily eliminated via the kidneys depends on the severity of renal dysfunction, because antimicrobial clearance is associated with Ccr [42, 59]. In patients with mild to moderate renal dysfunction (Ccr < 50 mL/min), these antimicrobials may require dose reduction, the need for which depends on the tolerability and safety of the agents. The approach to dose adjustment depends on whether the antimicrobial is concentration-dependent or time-dependent. For concentration-dependent antimicrobials such as aminoglycosides and daptomycin, extending the dosing interval without changing the single-dose amount would generally be preferable to maximize the Cpeak/MIC. Conversely, for time-dependent antimicrobials such as β-lactams, reducing the single-dose amount without altering the dosing interval would be optimal to maximize the fT > MIC. Several antimicrobials, particularly aminoglycosides and vancomycin, have been found to cause acute renal tubular toxicity in a concentration-dependent manner. If exposure to these antimicrobials increases during AKI and reaches the toxic window, they may themselves cause renal damage, leading to a negative spiral of AKI. Therefore, a careful dosing design based on more accurate assessment of renal function is required, especially when using nephrotoxic antimicrobials in patients with AKI. Serum creatinine, the gold standard for evaluating renal function, is the end product of metabolism of creatine, an amino acid that serves as an energy source for muscle contraction. Serum creatinine levels are sensitive to muscle mass, leading to overestimation of creatinine-based estimated glomerular filtration rate (eGFR) in patients who are malnourished or severely emaciated. In addition, increase in serum creatinine level during AKI lags behind a decrease in GFR, which may result in underestimation of renal function. Cystatin C, a low molecular weight protein produced at a constant rate in nucleated cells throughout the body, can also be used to estimate renal function [60]. Cystatin C is considered more accurate than creatinine for estimating renal function in patients with extremely low muscle mass and in elderly patients [61]. Several studies and a meta-analysis have also demonstrated the significance of cystatin C as an early diagnostic indicator for AKI [62–64]. Therefore, to determine antimicrobial dosing regimens during AKI, it would be advisable to assess the renal function by considering the changes in both creatinine-based eGFR and other indicators including cystatin C-based eGFR and urine volume.

### Renal replacement therapy

RRT is a treatment modality that removes waste products and urinary toxins from the blood, adjusts electrolytes and acid-base equilibrium, and corrects body fluids through dehydration. This therapy supports renal function and removes inflammatory cytokines and mediators in acute conditions such as sepsis and multi-organ failure. Therefore, critically ill patients frequently undergo RRT, especially CRRT, as they are often complicated with AKI and sepsis. CRRT is primarily classified into three modalities: continuous venovenous hemofiltration (CVVH), continuous venovenous hemodialysis (CVVHD), and continuous venovenous hemodiafiltration (CVVHDF). These modalities can also remove antimicrobials with certain characteristics such as small molecular weight, low plasma protein binding, and low volume of distribution [65], by diffusion and/or convection mechanisms. A comparison of plasma concentrations for amikacin [66], ciprofloxacin [67], and linezolid [68] revealed no significant differences between CVVHDF and CVVHF, suggesting no difference in CRRT clearance between modalities. CRRT clearance can be calculated by multiplying the effluent flow rate (defined as the sum of dialysate and filtrate flow rates) by the free fraction [69]. Jamal et al. [70] demonstrated a positive correlation between effluent flow rate and extracorporeal clearance for vancomycin, meropenem, and piperacillin, suggesting that effluent flow rate reliably predicts antibiotic clearance in critically ill patients. CRRT is also used in patients with residual renal function. In such case, it is advisable to consider both the patient’s renal clearance and CRRT clearance to avert subtherapeutic antimicrobial exposure. Nonoshita et al. [71] developed a doripenem population PK model for ICU patients undergoing CRRT. By including the patient’s renal clearance and CRRT clearance in total clearance in this model, the objective function value was significantly reduced. However, eGFR and estimated creatinine clearance cannot distinguish between renal and CRRT clearances, because serum creatinine is removed by CRRT due to its small molecular weight. These renal function metrics may approximate total clearance for drugs that are primarily eliminated via the kidneys, which could be useful for dose design. Several CRRT membranes such as polyacrylonitrile (PAN), polymethyl methacrylate (PMMA), and acrylonitrile-sodium methallylsulfonate copolymer (AN69ST) are capable of adsorbing and removing cytokines and other substances through hydrophobic or ionic bonding. These adsorbent membranes can bind several antimicrobials including vancomycin, gentamicin, ciprofloxacin, tigecycline, and teicoplanin [72–74], which may result in subtherapeutic plasma levels and impact patient outcomes. Vancomycin has been reported to adsorb to PAN membranes, to an extent that is significant enough to warrant consideration of dose adjustment during CRRT [72, 74]. Gentamicin and tigecycline exhibit significant adsorption on PAN membranes, with a substantial portion of the administered dose lost to adsorption [72]. Shiraishi et al. [73] evaluated and compared the adsorption rates of linezolid, vancomycin, and teicoplanin onto polysulfone (PS), PAN, and PMMA membranes. Their study demonstrated that teicoplanin had the highest adsorption rate across all membranes, with higher rates observed for PS and PMMA membranes than PAN. However, these in vitro experiments did not provide optimal dose regimen for each membrane. Although adsorption may play a critical role in the PK of antimicrobials during CRRT, it would be preferable to consider the overall PK of antimicrobials, including clearance rates and distribution volumes. Consequently, predicting antimicrobial concentrations during CRRT is challenging due to the influence of multiple factors. Therefore, TDM is recommended when available, especially for antibiotics, because subtherapeutic exposure may lead to treatment failure and antimicrobial resistance.

### Extracorporeal membrane oxygenation

ECMO is a critical life support technique used to provide temporary cardiopulmonary support for critically ill patients with severe heart and lung failure. It functions by circulating blood through an artificial lung outside the body, which adds oxygen to and removes carbon dioxide from the blood. The ECMO machine contains a variety of components within the circuit, such as a pump, cannula, tube, and membrane. As both veno-venous and veno-arterial ECMO configurations require a circuit with a large surface area, this can also significantly impact the PK of antimicrobials in critically ill patients by increasing the volume of distribution and decreasing clearance [75]. Circuit components and coatings may also adsorb antimicrobials, thereby reducing plasma concentrations. In particular, lipophilic and highly protein-bound agents are more susceptible to sequestration within the circuit [75, 76]. Sequestration may decrease over time as the ECMO circuit becomes saturated, which may also result in drug release from the ECMO circuit after drug discontinuation. A systematic review by Jendoubi et al. [77] identified 36 studies, including ex-vivo experiments and clinical studies, demonstrating significant sequestration of certain lipophilic and protein-bound antifungals including voriconazole, posaconazole, and micafungin, within the ECMO circuit. In contrast, Destache et al. [78] found higher cefepime concentrations during ECMO compared to pre-ECMO levels. These studies suggest that ECMO therapy may result in subtherapeutic and elevated serum concentrations of antimicrobials. Kim et al. [79] provide a thorough review of the PK/PD of antimicrobials in critically ill patients undergoing ECMO, highlighting the challenges in determining the optimal dosing strategy for these patients. One potential reason for this challenge is that most patients receive CRRT in addition to ECMO, which makes it impossible to evaluate the impact of ECMO alone. The review article underscores the importance of dose tailoring through TDM to personalize antimicrobial therapy in the setting of ECMO.

### Therapeutic drug monitoring in critically ill patients

#### Significance of proactive therapeutic drug monitoring for linezolid and β-lactams

For critically ill patients, antimicrobial dosing regimens should be determined based on the understanding of the PK variability described above. However, such populations frequently exhibit diverse and multiple sources of PK variability, posing challenges to develop robust dosing regimens. TDM is an excellent solution to this problem, as it provides individualized dosing for each patient using measured concentrations. A position paper from four related international societies recommended routine TDM for vancomycin, teicoplanin, aminoglycosides, linezolid, β-lactams, and voriconazole in critically ill patients [80]. The target therapeutic window for linezolid TDM ranges from 2 to 7 mg/L, as stated in the position paper [80], which is consistent with the ranges reported in other review articles [81, 82]. A prospective, open-label, interventional study evaluated the effectiveness of linezolid TDM intervention, suggesting the potential benefit of proactive linezolid TDM in preventing or recovering from dose-dependent thrombocytopenia. However, it should be noted that TDM for linezolid and β-lactams is not currently covered by medical insurance in Japan, as of February 2025. For β-lactams, the target PK/PD index of over 100% fT > MIC (trough concentration above MIC throughout dosing interval) in critically ill patients is consistent across the drug class [80, 82]. For several β-lactams, the trough concentration has been linked to neurotoxicity, such as seizures, with reported minimum thresholds of 20 mg/L for cefepime [83], 45 mg/L for meropenem [84], and 361 mg/L for piperacillin [85]. A meta-analysis and systematic review evaluated the benefit of TDM-guided dosing for β-lactams using four identified randomized control trials and seven retrospective studies [86]. The results showed that TDM-guided dosing significantly improved clinical and microbiological efficacy and reduced treatment failure, although there was no significant difference in mortality. Based on the evidence presented, proactive TDM is recommended for linezolid and β-lactams, in addition to vancomycin, teicoplanin, aminoglycosides, and voriconazole for critically ill patients in Japan.

#### Model-informed precision dosing

Model-informed precision dosing (MIPD) is an emerging framework that integrates multiple data sources to facilitate TDM and maximize the success of antimicrobial therapy [87]. Traditional TDM measures drug concentrations at steady state and evaluates whether they are within the target therapeutic window, followed by adjusting the dose based on the experience of clinicians or pharmacists. While this approach has the advantages of enhancing accessibility and simplifying interpretation of measurement results, it also has several drawbacks. The timing of blood sampling, such as trough and peak, is crucial, and typically requires waiting until the steady state is attained. In addition, the extent of drug exposure may vary depending on the number of doses and patient background such as renal function, even though the trough value remains constant. As a result, a single concentration, such as the trough value, may not be an optimal surrogate indicator. In contrast, MIPD provides an individualized dosing regimen that can achieve target PK/PD indices, based on multiple covariates prior to the first dose. Blood sampling before steady state is possible. After Bayesian estimation, individual dosing regimens can be optimized based on patient-specific PK parameters to maximize the probability of achieving the target PK/PD indices. However, the model used should be selected carefully, taking into account factors such as age group, indications, medical conditions (general ward, critically ill), body composition (normal, obese, cachexia), genetic status, dose and covariate ranges considered, and analytical methods employed. It should also be noted that the models are often not developed specifically for MIPD, and external validation of the predictive performance may not be adequate [88]. MIPD has been primarily used in clinical settings to guide vancomycin dosing. This approach will be proactively expanded to other antimicrobials, particularly for critically ill patients.

## Conclusions

This review article provides a comprehensive overview of factors that potentially influence the PK of antimicrobials and the effectiveness of TDM in critically ill patients. The dosing regimen of antimicrobials should be determined to ensure target PK/PD indices. However, the prediction of antimicrobial PK in critically ill patients is complicated by a complex interplay of multiple factors including inflammation, ARC, hypoalbuminemia, AKI, CRRT, and ECMO. TDM is the most effective approach to address this issue, but it is available for a limited number of antimicrobials in Japan. In recent years, the need for TDM, especially for β-lactams and linezolid, has received increasing attention. The introduction of MIPD for these antimicrobials is expected to promote personalized medicine for critically ill patients and improve the survival of these patients.

## Data Availability

No datasets were generated or analysed during the current study.

## Abbreviations

AKI: Acute kidney injury
ARC: Augmented renal clearance
AUC: Area under the curve during 24 h
Ccr: Creatinine clearance
Cpeak: Peak plasma concentration
CRP: C-reactive protein
CRRT: Continuous renal replacement therapy
CSF: Cerebrospinal fluid
CYP: Cytochrome P450
CVVH: Continuous venovenous hemofiltration
CVVHD: Continuous venovenous hemodialysis
CVVHDF: Continuous venovenous hemodiafiltration
ECMO: Extracorporeal membrane oxygenation
eGFR: Estimated glomerular filtration rate
FN: Febrile neutropenia
fT > MIC: Cumulative percent of time in 24 h that free drug concentration remains above minimum inhibitory concentration
ICU: Intensive care unit
IL: Interleukin
MIC: Minimum inhibitory concentration
MIPD: Model-informed precision dosing
PAN: Polyacrylonitrile
PD: Pharmacodynamic
PK: Pharmacokinetic
PMMA: Polymethyl methacrylate
PS: Polysulfone
RRT: Renal replacement therapy
SIRS: Systemic inflammatory response syndrome
TDM: Therapeutic drug monitoring
